# Buyers, Maybe Moving Second Is Not That Bad After All: Low-Power, Anxiety, and Making Inferior First Offers

**DOI:** 10.3389/fpsyg.2021.677653

**Published:** 2021-05-31

**Authors:** Yossi Maaravi, Ben Heller

**Affiliations:** ^1^The Adelson School of Entrepreneurship, Interdisciplinary Center, Herzliya, Israel; ^2^Baruch Ivcher School of Psychology, Interdisciplinary Center, Herzliya, Israel

**Keywords:** first offer, power, BATNA, negotiation, first-mover advantage, second-mover advantage, anchoring and adjustment

## Abstract

The behavioral decision-making and negotiations literature usually advocates a first-mover advantage, explained the anchoring and adjustment heuristic. Thus, buyers, who according to the social norm, tend to move second, strive to make the first offer to take advantage of this effect. On the other hand, negotiation practitioners and experts often advise the opposite, i.e., moving second. These opposite recommendations regarding first offers are termed the *Practitioner-Researcher paradox*. In the current article, we investigate the circumstances under which buyers would make less favorable first offers than they would receive were they to move second, focusing on low power and anxiety during negotiations. Across two studies, we manipulated negotiators' best alternative to the negotiated agreement (BATNA) and measured their anxiety. Our results show that, when facing neutral-power sellers, weak buyers who feel anxious would make inferior first offers (Studies 1 and 2). When facing low-power sellers, weak buyers would make inferior first offers across all anxiety levels (Study 2). Our findings shed light on two critical factors leading to the Practitioner-Researcher paradox: power and anxiety, and offer concrete guidelines to buyers who find themselves at low power and highly anxious during negotiations.

## Introduction

In most negotiations, buyers are at an inherent disadvantage due to the social norm of sellers making the first move (Maaravi et al., 2014). By making the first move, sellers benefit from the “anchoring” effect. Indeed, research has long established that negotiators' first offers are “anchors” (Tversky and Kahneman, 1974) that affect both counteroffers and settlement prices (e.g., Galinsky and Mussweiler, 2001). But buyers can avoid this inherent disadvantage by making the first offer themselves, thereby establishing a more favorable anchor resulting in a better settlement price. But is this advice to sellers necessarily beneficial? Interestingly, recent findings point to specific situations where negotiators make *disadvantageous* first offers than the ones they could have received from their counterparts (Maaravi and Levy, 2017). These new studies were labeled the *Practitioner-Researcher Paradox* since they describe what negotiation experts—as opposed to negotiation scholars—long believe: sometimes, it is better *not* to make the first offer (Loschelder et al., 2016).

Continuing this innovative research, the current studies explored the circumstances under which buyers would make less favorable first offers than they would have received were they to move second. Specifically, building on past research that has established the importance of power (Schaerer et al., 2015) and anxiety (Brooks and Schweitzer, 2011) in negotiation, we investigated the effects these factors might have on buyers' first offers.

### Literature Review

The past two decades have seen an upsurge in research into the determinants and consequences of first offers in negotiations (e.g., Ames and Mason, 2015; Maaravi and Hameiri, 2019). Previous research has established that first offers determine settlement prices through their anchoring effect (Tversky and Kahneman, 1974) on counteroffers (Galinsky and Mussweiler, 2001), such that the counteroffer and consequent settlement price are closer to the first offer. Since this robust finding is symmetrical for both sellers and buyers, buyers are commonly advised to make the first offer to compensate for their inherent disadvantage (Malhotra and Bazerman, 2007).

However, a growing body of research points to the possible limitations of offering first. For example, initiators may suffer from lower satisfaction at the end of the negotiation and heightened anxiety levels before and during the negotiation (Rosette et al., 2014). Another study found that learning and using the anchoring strategy (move first, be extreme) led to worse long-term psychological and economic results (Maaravi et al., 2014). In this research, counterparts were less satisfied with their results and thus less willing to negotiate with the first-mover in the future. In market settings investigated in the same study, first-movers made lower overall profits due to prolonged negotiation processes and more impasses.

Of greater relevance is the recent investigation line that has begun to doubt the general first-mover advantage. Two factors were researched within this context: (1) revealing private information about compatible preferences, which the recipients could take advantage of (Loschelder et al., 2014); and (2) asymmetry of information which may cause negotiators to make worse first offers than those of their opponents (Maaravi and Levy, 2017).

A critical negotiation-related variable is *power*, mainly determined by negotiators' alternatives (BATNA, i.e., Best Alternative to a Negotiated Agreement, Bazerman and Neale, 1993). Power has been found to impact the process and the final result of negotiations (Magee et al., 2007; Schweinsberg et al., 2012). Thus, it has become a central subject of negotiation research (Schaerer et al., 2015). For example, merely thinking about counterpart's BATNA impacted first offers, counteroffers, and settlement prices (Galinsky and Mussweiler, 2001). Additionally, increasing negotiators' perceived power, either by priming cues or providing good alternatives, led to increased amounts of the first offers, resulting in better outcomes (Magee et al., 2007).

But if greater negotiation power leads to more beneficial first offers, low power may have the opposite result. Imagine, for example, a buyer who negotiates with the owner (A) of a used car and has one other alternative—the owner of car B. Not knowing A's expectations, if the buyer makes the first offer, she might use B's price as a reference point (or anchor) and adjust from it. If the price ranges between 10,000 to 15,000$ and B has asked for 16,000$, the buyer—self-anchored by the 16,000$–might offer A 14,000$. But, if A, who has weak alternatives of his own, planned on asking 12,000$, the buyer's first offer is not optimal. Similarly, Schaerer et al.'s (2015) have shown that lack of alternatives may be better than mediocre ones since they anchor the initiator to make a modest first offer. Here, we expand this reasoning and argue that low power buyers would make inferior first offers.

Notwithstanding the norm of sellers making the first move, there are circumstances in which buyers have the opportunity to deviate from this norm. Examples include salary negotiations (Magee et al., 2007), professional athletes' contracts (Loschelder et al., 2014), angel investments in startups (Bammens and Collewaert, 2014), or selling of real estate assets or special items such as antiques (Maaravi and Levy, 2017). Thus, it is beneficial for buyers to understand the circumstances in which they would make less favorable first offers so that they actively and strategically decide whether to abide by the norms (move second) or not.

Another variable that is relevant in this context is *anxiety*. Given the uncertainty in negotiations, people usually experience high anxiety levels (Small et al., 2007). This finding is in line with anxiety-uncertainty management theory (Gudykunst, 2005) and the definition of anxiety as “*a state of distress and/or physiological arousal in reaction to stimuli including novel situations and the potential for undesirable outcomes”* (Brooks and Schweitzer, 2011). This is amplified when negotiators make the first offers. Indeed, feelings of anxiety decreased initiators' satisfaction with their results, although these results were economically better than second-movers results (Rosette et al., 2014). Of greater relevance is the research by Brooks and Schweitzer (2011), which showed that anxious negotiators expected lower results, made lower first offers, reached worse settlement prices, etc. Thus, we would expect to find an effect of anxiety on buyers' first offers. Yet our study provides the possibility of testing a unique interaction: not only do negotiations elicit anxiety, but low-power negotiators would experience even greater anxiety. This interaction could lead to differing effects of low power depending on the degree of experienced anxiety.

### Hypotheses and the Current Research

To summarize, our hypotheses were as follows:

(H1) Buyers with low power (weak BATNAs) would receive a more favorable offer were they to move second in the negotiation (compared to the first offer they would have made themselves);

(H2) Anxiety would moderate this effect, such that more anxious weak-BATNA buyers would make even less favorable first offers compared to their less anxious counterparts.

## Study 1

In Study 1, we used a basic design to test whether low-power buyers would make less favorable first offers.

### Methods

#### Participants

One hundred and eleven participants (54 males, 56 females, 1 other; average age: 42.59, *SD* = 10.66) were recruited via Amazon's “Mechanical Turk” (MTurk) crowd-working platform and compensated for participation. MTurk samples tend to be marginally more diverse than other types of internet samples yet are similarly reliable (Paolacci et al., 2010; Buhrmester et al., 2011).

#### Design

The between-groups design varied power between two different roles. Participants were randomly assigned to either low power (weak BATNA) buyer or neutral (no BATNA) seller conditions. The dependent variable was the first offer made by participants. Within-negotiation anxiety was measured as a moderating variable.

#### Procedure

Our procedure was based on the used car scenario used by Kwon and Weingart (2004). In the buyer condition, participants were avid car collectors interested in renovating an old car. They read that they saw an ad for a car of the same model up for sale. To further emphasize their low power, BATNA information said that their *only* alternative was to buy the parts individually, which could cost $3,700. They were then informed that they decided to approach the seller to buy the car and were required to state their initial proposal. To provide market price information, the scenario read that similar cars' prices ranged between $1,200 and $2,000.

In the seller condition, participants were asked to imagine that they were looking into selling their grandma's old car. Hence, they published a newspaper ad and were told that they had received a few offers ($1,000–$1,300). They, too, were given the same market price information and were told that a new buyer had just called and that they were about to meet him. They were then asked to state their initial demand.

#### Measures

After reporting their first offer, participants filled out the “state” items of the State-Trait Anxiety Inventory (STAI; Spielberger, 2010). This questionnaire comprises 20 items measuring an individual's self-reported anxiety *at a particular moment*, evaluating qualities such as a person's feelings of tension, nervousness, or worry. It is one of the most validated and widely used state anxiety questionnaires. As such, we used it to measure the anxiety participants had experienced when reading the scenario and stating their first offer.

### Results

The STAI was found to be highly reliable (α = 0.95). An independent-samples *t*-test compared the first offer for the weak-buyer and neutral-seller conditions. There was a significant difference in first offer between weak-buyer (*M* = 1,415.17, *SD* = 294.01) and neutral-seller (*M* = 1,710.90, *SD* = 256.52) conditions: *t*_(109)_ = −5.64, *p* < 0.001. In other words, and contra to our hypothesis (H1), low power buyers would have made significantly lower first offers than what the sellers demanded, which according to past research, would result in an unfavorable anchoring effect on future counteroffers and the settlement price.

**Figure 1:**
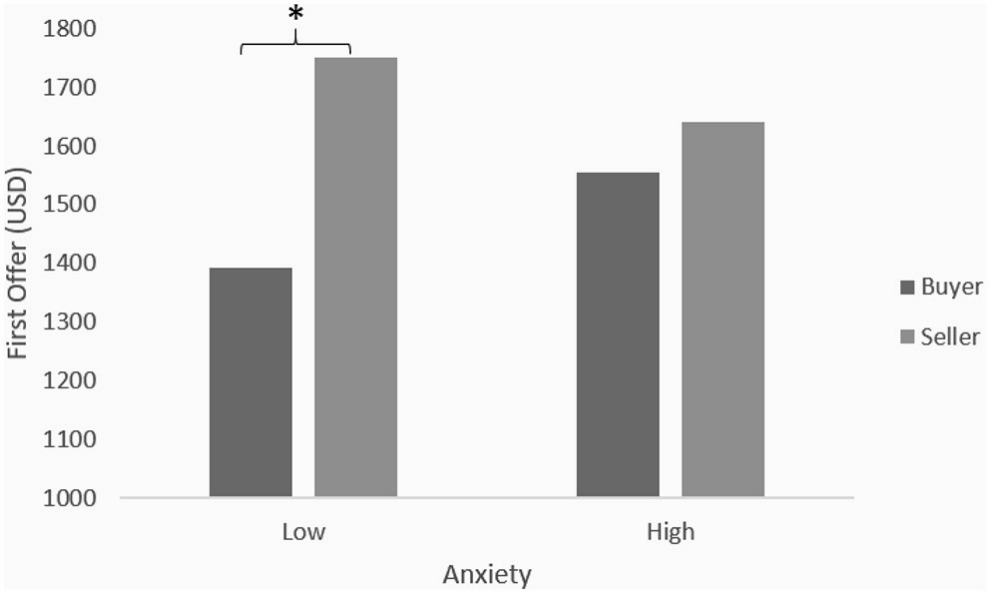
Mean first offer—ford thunderbird negotiation. *p < 0.05.

Anxiety was examined as a moderator of the relation between power and first offer. Anxiety and power were entered in the first step of the regression analysis. No main effect for anxiety was found (β = 0.005, *B* = 2.04, *t* = 0.06, ns). In the second step of the analysis, the interaction between anxiety and power was entered, revealing a trend in the expected direction that fell short of statistical significance (β = −0.16, *B* = −129.4, *t* = −1.94, *p* = 0.055). The increase in variance in first offer [Δ*R*^2^ = 0.026, *F*_(1,107)_ = 3.77, *p* = 0.055] shows a similar trend that did not reach statistical significance. Tests of simple slopes indicated that role had a significant effect on the first offer when anxiety was low (β = 0.58, *B* = 365.66, *t* = 5.78 *p* < 0.001), but not when anxiety was high (β = 0.17, *B* = 106.86, *t* = 0.96, ns). As shown in Figure 1, when anxiety was high, there was no significant difference between weak buyers' and neutral sellers' first offers.

That is, merely having a weak alternative did not necessarily mean buyers should prefer to move second, as their first offers were significantly lower than those of sellers. But, anxious buyers with weak alternatives should choose to move second as their offers were almost identical to those of buyers. In this case, letting sellers move first would allow buyers to bargain and *decrease* the price instead of making the same offer themselves and allowing sellers to negotiate and *increase* it.

Although these results point toward less favorable first offers among anxious buyers with weak BATNAs, Study 1 used a rather basic setup, was exploratory, and had a few limitations. First, it only compared weak buyers with no-BATNA sellers. Based on past research that has established that strong BATNA negotiators benefit from making the first offer (Galinsky and Mussweiler, 2001), and taking into account the results of Study 1, we can expect to confirm H1 when weak buyers face weak sellers. Second, Study 1 used a basic scenario that only described the range of prices and the alternative price (BATNA). It is essential to test our hypotheses using a more complex scenario with additional market information. Finally, Study 1 was conducted among American “Mturk” participants. To generalize our results, it is crucial to use a culturally different sample. Study 2 was conducted to expand these initial findings and address the critiques mentioned above.

## Study 2

After establishing that, when anxious, weak-BATNA buyers make unfavorable first offers, we conducted Study 2 to test this result across another more complex negotiation scenario, additional BATNA combinations, and a different population.

### Methods

#### Participants

One hundred seventy-one participants (86 males and 85 females; average age: 44.31, *SD* = 15.11) were recruited via the “Midgam Panel” and compensated for participation. The “Midgam Panel” is an Israeli crowd-working platform similar to MTurk (Huff and Tingley, 2015).

#### Design

We used a 2 (weak BATNA vs. no BATNA) ^*^ 2 (seller vs. buyer) between-groups design. The dependent variable was the first offer made by the participants. Within-negotiation anxiety was measured as a possible moderating variable.

#### Procedure

We used an adapted version of the pharmaceutical factory paradigm used by Galinsky and Mussweiler (2001). This scenario is commonly used in research on distributive negotiations (e.g., Galinsky et al., 2005; Maaravi et al., 2011; Kang et al., 2015).

In the buyer conditions, participants imagined that they were a CEO of a pharmaceutical company looking to buy a factory to manufacture a line of highly specialized compounds. To this end, they were about to start negotiations with a seller of one such factory and were given the following information: the plant for sale was located in an area that contained many startup biotechnology firms and an experienced and highly mobile workforce; the factory was bought 3 years earlier for 15 million NIS; the factory was estimated to be worth around 19 million NIS 2 years ago; a similar factory was recently sold for 26 million NIS. In the neutral power condition, participants read that their only other alternative was to build a new factory, which included buying the equipment and hiring a skilled workforce. In the low power condition, participants read that this alternative's cost (building a new factory) was estimated to be around 32 million NIS. They were then required to state their first offer in the current negotiation.

In the seller condition, participants read that they were the owners of a pharmaceutical factory looking to sell it because they were phasing out their current product line. They were given the same general information that the buyer condition received and were told that they were about to start negotiations with a potential buyer. In the neutral power condition, participants read that their only alternative was to dismantle the factory, which would mean selling the land and the equipment separately. In the low power condition, participants read that selling in this manner would reap them around 8 million NIS. They were then required to state their first offer in the current negotiation.

All scenarios and questions were written in the Hebrew language.

#### Measures

After reporting their first offer, participants filled out the same anxiety measure used in Study 1, except this time, we used the Hebrew language version of the questionnaire.

### Results

Again, the state anxiety inventory was found to be highly reliable (α = 0.95).

**Figure 2:**
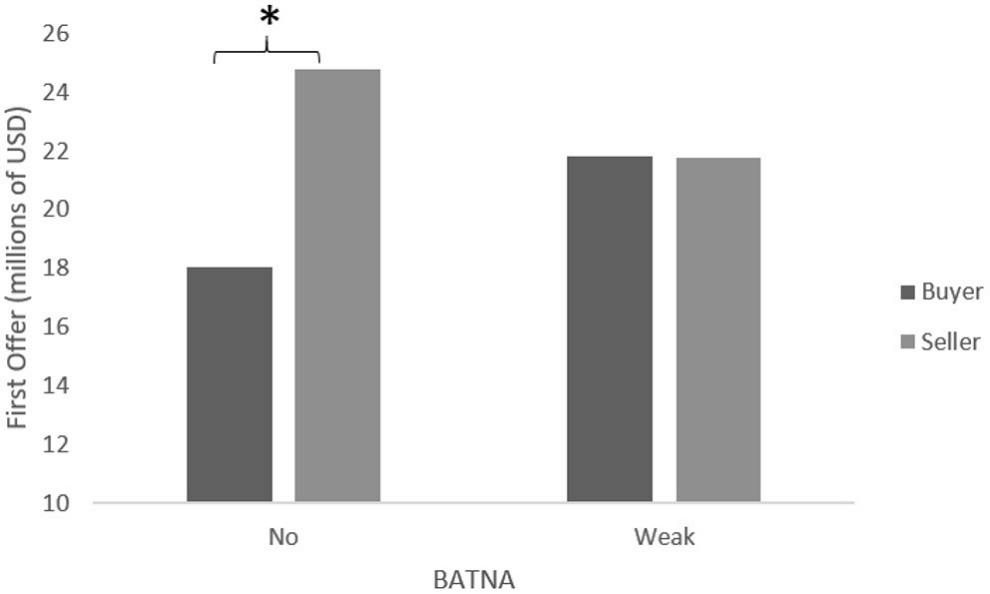
Mean first offer—pharmaceutical plant negotiation. *p < 0.05.

A two-way ANOVA was conducted to examine the effect of role and power on the first offer. There was a statistically significant interaction between the effects of role and power on the first offer, *F*_(1,169)_ = 20.49, *p* < 0.001. Simple main effects analysis showed that buyers and sellers differed significantly in first offers (Mbuyers = 18.02; Msellers = 24.84; *p* < 0.001) under the neutral power conditions. There were no differences between buyer and seller first offers under the low power conditions (Mbuyers = 21.8; Msellers = 21.74; ns), as shown in Figure 2. In other words, when both buyers and sellers had low power, their first offers were the same, which, supporting H1, implies that their moving first would result in a more favorable anchor.

Additionally, we ran moderation analyses on these significant effects with anxiety as the moderator, as per our hypothesis (H2). Anxiety was examined as a moderator of the relation between power and first offer in two different analyses: (1) low power buyer vs. neutral seller; and (2) neutral buyer vs. low power seller. Note that, without examining the moderating effect of anxiety, these dyads yielded significant differences in the first offer, such that each negotiator would make a more favorable first offer: *t*_(88)_ = −2.92, *p* < 0.01 and *t*_(79)_ = −3.30, *p* < 0.001, respectively. Importantly, we did not run a moderation analysis on the weak buyer vs. weak seller comparison. The above results imply that buyers should move second to establish a more favorable anchor.

**Figure 3:**
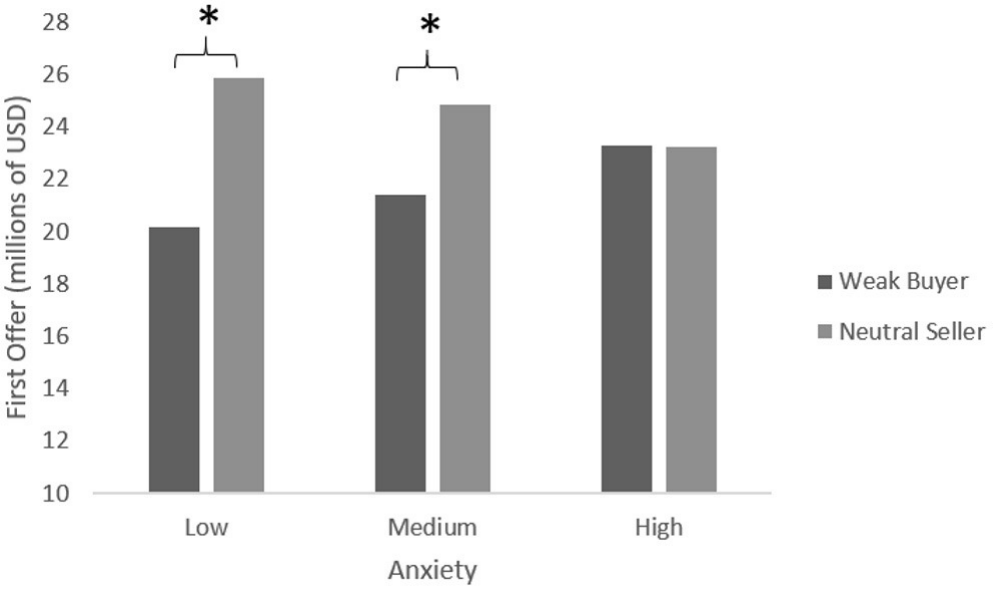
Mean first offer—differing levels of anxiety. *p < 0.05.

In the low power buyer vs. neutral seller comparison, anxiety and power were entered in the first step of the regression analysis. A main effect for anxiety was found (β = 0.53, *B* = 0.23, *t* = 2.55, *p* = 0.012). In the second step of the regression analysis, the interaction term between anxiety and power was entered revealing a significant effect (β = −0.25, *B* = −0.11, *t* = −2.51, *p* = 0.013) which explained a significant increase in variance in first offer, Δ*R*^2^ = 0.069, *F*_(1,86)_ = 6.31, *p* = 0.013. Tests of simple slopes indicated that power had a significant effect on the first offer when anxiety was low (β = 0.57, *B* = 2.86, *t* = 3.93, *p* = 0.002) or medium (β = 0.34, *B* = 1.73, *t* = 3.43 *p* = 0.009), but did not have an effect when anxiety was high (β = −0.004, *B* = −0.02, *t* = −0.03, ns). In other words, and as can be seen in Figure 3, when anxiety was high, there was no significant difference between low power buyers' and neutral sellers' first offer. This result is in line with H2.

In the neutral power buyer vs. weak seller comparison, on the other hand, anxiety was not found to be a significant moderator of the effect of power on first offers when examining the effect between neutral buyers and low power sellers.

An interesting contribution of this study was in the case when both negotiators had weak alternatives. In this case, the first offers did not differ significantly. As in the previous study's conclusion, this would render it more favorable for buyers to move second due to their subsequent ability to move the negotiation in their favor. Additionally, the anxiety findings in this study also replicated Study 1, but only for low power buyers. In other words, given that their first offer would be equal to their counterparts, highly anxious buyers should strive to move second due to their ability to move the negotiation in their favor via their counteroffer. Interestingly, this finding was not found for low power sellers, meaning that highly anxious low power sellers would make a more favorable first offer, which according to the literature, should result in a better settlement price. This asymmetry, which has been established in past research (Maaravi et al., 2014), may be due to sellers' inherent power attributed to the endowment effect (Morewedge et al., 2009).

## General Discussion

Although most negotiation scholars recommend that buyers counteract their inherent disadvantage by making the first move in negotiations (due to anchoring effects; Galinsky and Mussweiler, 2001), this may not always be the favorable course of action. Specifically, and based on the relationship between first offers and outcomes of negotiations, we examined the effects of two variables present in most negotiations: power and anxiety. We hypothesized that: (1) low power buyers would make a less favorable first offer than they would have received were they to move second; and (2) negotiators' anxiety would moderate this effect; such that more anxious negotiators would make even less beneficial first offers. This research can provide further insight into the Practitioner-Researcher Paradox (Loschelder et al., 2014) and explain why some negotiation experts recommend that negotiators move second.

First, and contra to our hypothesis, we found that low power buyers in negotiations with a neutral power (no BATNA) seller made more favorable first offers than they would have received were they to make the counteroffer. Nevertheless, and in partial support of our hypothesis, we found that when both sides of the negotiation (buyers and sellers) had low power, buyers and sellers made identical first offers (Study 2). Therefore, second-movers could have benefited from making the counteroffer by moving the negotiation in their favor. Interestingly, when buyers had neutral power, their first offers were more favorable than those they would have received (moving second). This finding echoes a recent finding, which showed that having no power is better than having low power (Schaerer et al., 2015). Second, the results of both studies showed that when low power buyers were highly anxious, their first offers were equal to those of their neutral power counterparts, confirming our hypothesis regarding anxiety's moderating effect. As in our previous conclusion, when both sides' first offers are the same, they should strive to move second due to their ability to steer the negotiation in their favor. Interestingly, this pattern did not repeat itself for low power sellers negotiating with neutral power buyers, a finding which will be discussed below.

These results and their consequent implications add to the emerging literature showing the precursors and circumstances leading to a first-mover disadvantage (Maaravi et al., 2014; Rosette et al., 2014). Our findings suggest that buyers should be aware of their alternatives and their level of anxiety. Specifically, anxious buyers with weak alternatives should strive to make the counteroffer, given the anchoring effects of first offers on outcomes. Alternatively, they should consider adopting precautionary anti-anchoring strategies (e.g., perspective-taking, Galinsky and Mussweiler, 2001) or apply anxiety-reducing techniques (e.g., taking a neutral stance, clearing one's mind; Wheeler, 2004) before making the first offer.

Our study is not without its limitations. First, across both of our studies, participants were either given a weak BATNA (low power condition) or no BATNA information at all (neutral power condition). We chose to leave high power negotiators out because of the vast amount of previous research which specifically focused on them (e.g., Magee et al., 2007). Although our hypotheses revolved around low power negotiators, future studies should measure buyers' first offers with medium or high power (strong BATNA) and compare them with their low power counterparts. This will shed light on whether anxious, low power buyers should strive to move second when negotiating with not only low or neutral power counterparts but possibly also medium power ones.

Second, in light of the current research's exploratory nature, the negotiation scenario across both studies culminated in the negotiators' first offers, and participants did not complete a whole negotiation. Due to this limited procedure, we cannot draw explicit conclusions about how anxiety and low power interact to affect the latter stages of the negotiation, such as counteroffers and final settlement prices. Nevertheless, given the vast literature covering the relationship between first offers and outcomes, we suspect that future studies examining the later stages of negotiations should replicate our findings.

Third, our study may suffer from generalizability issues due to the lack of incentives to achieve a better outcome and unfamiliar negotiation scenarios. Nevertheless, incentive-less experimental paradigms are standard practice in negotiation research (e.g., Gunia et al., 2013), and our scenarios have a basis in previously published studies (Kwon and Weingart, 2004). Additionally, while multi-issue negotiations are more common, single-occurrence, single-issue transactions of the kind we use in our research (i.e., cars and property) occur throughout one's life. Thus, they are of interest to both business people and the general public. Nevertheless, future studies should seek to replicate our findings while using incentives for successful negotiations, along with more common and familiar negotiation scenarios.

Finally, negotiated outcomes depend heavily on the market conditions, especially on the “supply-demand” balance of the number of sellers vs. the number of buyers in the market. But, in line with past research (Magee et al., 2007), we only manipulated power through the *quality* of alternatives and not their *quantity*. Future research should also investigate market conditions in terms of the number of alternatives.

In conclusion, our findings demonstrate the adverse effects of high anxiety and low power on first offers. This study expands recent findings around the second-mover advantage and offers practical advice to buyers wishing to settle the “Researcher-Practitioner Paradox.”

## Data Availability Statement

The data supporting the findings of these studies are openly available at: https://osf.io/vxkpe/?view_only=a7e08e889c1c4dc09f02948787dea397.

## Ethics Statement

The studies involving human participants were reviewed and approved by the Institutional Review Board (IRB), IDC Herzliya. All subjects provided informed consent to participate in the study. To protect the respondents' privacy, the survey was conducted anonymously. All procedures were in accordance with the Declaration of Helsinki. The patients/participants provided their written informed consent to participate in this study.

## Author Contributions

YM: project administration, supervision, writing—review and editing, resources, methodology, and conceptualization. BH: visualization, writing-original draft, data curation, and formal analysis. All authors contributed to the article and approved the submitted version.

## Conflict of Interest

The authors declare that the research was conducted in the absence of any commercial or financial relationships that could be construed as a potential conflict of interest.

## Correction note

A correction has been made to this article. Details can be found at: 10.3389/fpsyg.2025.1652330.
