# Correction: Buyers, maybe moving second is not that bad after all: low-power, anxiety, and making inferior first offers

**DOI:** 10.3389/fpsyg.2025.1652330

**Published:** 2025-07-29

**Authors:** Yossi Maaravi, Ben Heller

**Affiliations:** ^1^The Adelson School of Entrepreneurship, Interdisciplinary Center, Herzliya, Israel; ^2^Baruch Ivcher School of Psychology, Interdisciplinary Center, Herzliya, Israel

**Keywords:** first offer, power, BATNA, negotiation, first-mover advantage, second-mover advantage, anchoring and adjustment

In the published article, there was an error in the text. Two *p*-values were rounded to two decimal places (instead of three) and consequently reported as statistically significant.

A correction has been made to **Study 1**, ***Results***, *Paragraph 2*.

This sentence previously stated:

“In the second step of the analysis, the interaction between anxiety and power was entered revealing a significant effect (β = −0.16, B = −129.4, t = −1.94, p = 0.05) which explained a significant increase in variance in first offer, ΔR^2^ = 0.026, F(1, 107) = 3.77, p = 0.05.”

The corrected sentence appears below:

“In the second step of the analysis, the interaction between anxiety and power was entered, revealing a trend in the expected direction that fell short of statistical significance (β = −0.16, *B* = −129.4, *t* = −1.94, *p* = 0.055). The increase in variance in first offer [Δ*R*^2^ = 0.026, *F*_(1,107)_ = 3.77, *p* = 0.055] shows a similar trend that did not reach statistical significance.”

Additionally, the correspondence email address as published has been amended. Instead of “myossi@idc.ac.il” it should be “myossi@runi.ac.il”.

The original article has been updated.

